# Effect of in-feed administration and withdrawal of tylosin phosphate on antibiotic resistance in enterococci isolated from feedlot steers

**DOI:** 10.3389/fmicb.2015.00483

**Published:** 2015-05-27

**Authors:** Alicia G. Beukers, Rahat Zaheer, Shaun R. Cook, Kim Stanford, Alexandre V. Chaves, Michael P. Ward, Tim A. McAllister

**Affiliations:** ^1^Faculty of Veterinary Science, The University of SydneySydney, NSW, Australia; ^2^Lethbridge Research Centre, Agriculture and Agri-Food CanadaLethbridge, AB, Canada; ^3^Alberta Agriculture and Rural Development, Lethbridge Research CentreLethbridge, AB, Canada

**Keywords:** enterococci, antimicrobial resistance, subtherapeutic macrolides, beef cattle, tylosin, erythromycin

## Abstract

Tylosin phosphate is a macrolide commonly administered to cattle in North America for the control of liver abscesses. This study investigated the effect of in-feed administration of tylosin phosphate to cattle at subtherapeutic levels and its subsequent withdrawal on macrolide resistance using enterococci as an indicator bacterium. Fecal samples were collected from steers that received no antibiotics and steers administered tylosin phosphate (11 ppm) in-feed for 197 days and withdrawn 28 days before slaughter. *Enterococcus* species isolated from fecal samples were identified through sequencing the *groES-EL* intergenic spacer region and subject to antimicrobial susceptibility testing, identification of resistance determinants and pulsed-field gel electrophoresis profiling. Tylosin increased (*P* < 0.05) the proportion of ery^R^ and tyl^R^ enterococci within the population. Just prior to its removal, the proportion of ery^R^ and tyl^R^ resistant enterococci began decreasing and continued to decrease after tylosin was withdrawn from the diet until there was no difference (*P* > 0.05) between treatments on d 225. This suggests that antibiotic withdrawal prior to slaughter contributes to a reduction in the proportion of macrolide resistant enterococci entering the food chain. Among the 504 enterococci isolates characterized, *Enterococcus hirae* was found to predominate (*n* = 431), followed by *Enterococcus villorum* (*n* = 32), *Enterococcus faecium* (*n* = 21), *Enterococcus durans* (*n* = 7), *Enterococcus casseliflavus* (*n* = 4), *Enterococcus mundtii* (*n* = 4), *Enterococcus gallinarum* (*n* = 3), *Enterococcus faecalis* (*n* = 1), and *Enterococcus thailandicus* (*n* = 1). The diversity of enterococci was greater in steers at arrival than at exit from the feedlot. Erythromycin resistant isolates harbored the *erm*(B) and/or *msrC* gene. Similar PFGE profiles of ery^R^ *E. hirae* pre- and post-antibiotic treatment suggest that increased abundance of ery^R^ enterococci after administration of tylosin phosphate reflects selection for strains that were already present within the gastrointestinal tract of cattle at arrival.

## Introduction

Subtherapeutic administration of antibiotics in livestock feed has come under increasing scrutiny due to concerns that such a practice increases the emergence of antibiotic resistant bacteria (Aarestrup, 1999). This concern is particularly relevant for bacteria that reside in livestock and are associated with clinical infections in humans.

Enterococci are commensal bacteria of the human and bovine gastrointestinal tract, but are also associated with nosocomial and community-acquired infections in humans (Poh et al., 2006; Franz et al., 2011). *Enterococcus faecalis* and *Enterococcus faecium* are the two species most frequently associated with enterococcal infections in humans, being responsible for as much as a third of the nosocomial infections worldwide (Werner et al., 2008). Whereas in cattle, *Enterococcus hirae*, a species not commonly associated with human infections is predominately isolated from bovine feces (Anderson et al., 2008; Jackson et al., 2010; Zaheer et al., 2013).

In North America, tylosin phosphate is commonly included in cattle feed for the control of liver abscesses (Page and Gautier, 2012). Previous research has shown therapeutic and subtherapeutic administrations of macrolides to cattle increases the proportion of erythromycin resistant enterococci in bovine feces (Jacob et al., 2008; Zaheer et al., 2013). In 2005, the WHO identified macrolides as critically important antimicrobials for which management strategies are urgently required to reduce the prevalence of bacterial resistance (Collignon et al., 2009). Macrolides are part of the MLS_B_ (macrolide-lincosamide-stretogramin B) superfamily with each antibiotic having slight structural differences, but resistance to one member of the family can cross-select for resistance to other drugs in the family. Consequently, if the inclusion of tylosin in feed leads to tylosin resistant enterococci in cattle it may also select for enterococci that are resistant to other macrolides such as erythromycin, an antibiotic important for the treatment of bacterial infections in humans (Roberts, 2008; Desmolaize et al., 2011).

Enterococci resistant to macrolides commonly carry the resistance determinant *erm*(B), an rRNA methlyase that confers cross-resistance to MLS_B_ antibiotics, or *msr*C, a macrolide efflux pump (Portillo et al., 2000). Very little is known about the nature and resistance characteristics of enterococci isolated from feedlot cattle. If *E. hirae* is consistently found as the predominant species in cattle feces, administering macrolides to cattle may not pose as a significant risk because this species is not commonly associated with human infections. Furthermore, antibiotics are often withdrawn prior to slaughter to reduce the risk of residues contaminating meat. In this study, we hypothesized that withdrawal of tylosin prior to slaughter would be an effective method of reducing the risk of resistant enterococci entering the food chain.

The objectives of this study were to determine the prevalence of macrolide resistant enterococci recovered from cattle continuously fed tylosin phosphate, and following its withdrawal. The recovered enterococci were characterized through species identification, antimicrobial susceptibility testing, identification of resistance determinants and pulsed-field gel electrophoresis (PFGE) profiling.

## Materials and methods

### Experimental design

The enterococci isolates investigated in this study were a subset of those archived during a larger study. Full methodological details have been described previously (Alexander et al., 2008; Sharma et al., 2008) and are summarized briefly below.

British crossbred steers (150 ± 20 kg) were randomly assigned to 10 pens (10 steers per pen) at the Lethbridge Research Centre feedlot (Lethbridge, Alberta, Canada). Steers were obtained from a single ranch (Deseret Ranches, Raymond, Alberta, Canada) and received no antibiotics prior to the beginning of the experiment.

**Figure 1:**
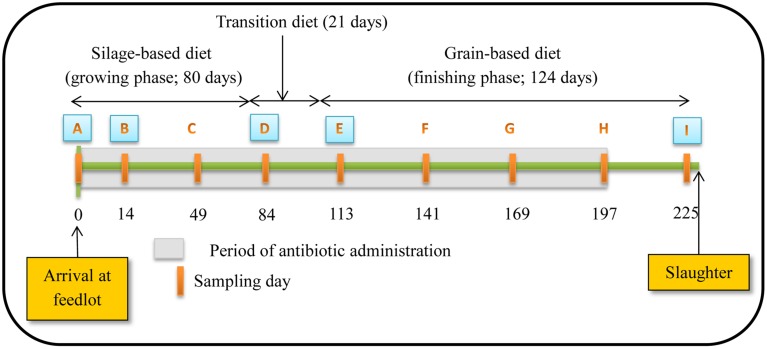
**Schematic representation of experiment timeline (Figure reproduced from Sharma et al., 2008)**. Numbers indicate day of feeding period. Periodic orange rectangles indicate points where fecal samples were collected from steers. A, B, D, E and I represent points where isolates were selected for assessing antibiotic susceptibility, PFGE profiles and identifying resistance determinants. Grey shaded area represents the period that tylosin was administered in the diet.

Five pens of cattle each were randomly assigned to one of two treatments: (i) control, no antibiotics (denoted CON); (ii) tylosin phosphate (Tylan®, Elanco Animal Health; treatment denoted T11) at 11 ppm in the diet. Tylosin was administered continuously for 197 days, starting on arrival at the feedlot and was withdrawn from the diet 28 days prior to slaughter (Figure 1). To avoid cross contamination between diets, tylosin was mixed with 5 kg of supplement and manually spread over the surface of the feed during the morning feeding. Steers were fed once daily to ensure that all feed allotted to each pen was consumed. Steers in CON and T11 treatments were housed in opposite sides of the feed alley to ensure that steers in different treatments did not have direct contact with one another. The animals involved in this study were cared for according to the guidelines set out by the Canadian Council on Animal Care (Canadian Council on Animal Care, 2003).

Steers were fed diets typical of the western Canadian feedlot industry during a growing and finishing period. For the growing period, a silage-based diet consisting of 70% barley silage, 25% barley grain, and 5% supplement on a dry-matter (DM) basis was fed for the first 80 days (Figure 1). Cattle were transitioned from the silage-based growing diet to a grain-based finishing diet (85% barley grain, 10% barley silage, and 5% supplement on a DM basis) over 21 days and maintained on this diet for a further 124 days until slaughtered. A common watering bowl was shared between adjacent pens on the same treatment.

### Sample collection and processing

The study occurred from November 2004 to July 2005. Rectal fecal samples were collected from each steer upon arrival at the feedlot and monthly thereafter until slaughter (Figure 1). Proportion of steers positive for macrolide resistant enterococci, CFU counts and the proportion of macrolide resistant enterococci in steers were estimated at all 9 sampling dates with enterococci isolates from 5 of these dates used for assessing antimicrobial susceptibility, identifying resistance determinants and PFGE profiles. The five sampling dates were selected to include isolates prior to administration of tylosin, during the growing and finishing feeding periods and post-withdrawal of tylosin from the diet.

On each sampling date, fecal grab samples were collected and immediately transported to the lab within 1 h after collection. At the lab, fecal slurries were created by mixing feces (10 g) with 90 ml of 1 × phosphate-buffered saline in a stomacher bag (Fisher Scientific, Ottawa, Ontario, Canada) and using a Stomacher (2 min, 230 rpm, room temperature; Seward Ltd., Worthing, West Sussex, United Kingdom). Slurries were serially diluted 10-fold and 100 μL of the appropriate dilution plated in duplicate onto Bile-Esculin-Azide (BEA; BD, Franklin Lakes, New Jersey, USA) agar containing no antibiotics or onto BEA amended with erythromycin (8 μg/mL; BEA^E^), or tylosin (32 μg/mL; BEA^T^) to select for enterococci resistant to erythromycin or tylosin. The breakpoint for erythromycin was based on the Clinical and Laboratory Standards Institute (CLSI) guidelines whilst an arbitrary value, based on avoiding plate over growth and the levels used by Davies and Roberts (1999), was selected for tylosin. Plates were incubated for 48 h at 37°C and colonies from BEA, BEA^E^, and BEA^T^ were enumerated. Two isolates from control plates and four isolates from antibiotic selective plates were streaked onto Trypticase soy agar (TSA; BD), incubated for 24 h, transferred to 20% glycerol in brain heart infusion broth (BD) and stored at −80°C until processed.

### Characterization of enterococci

A total of 1029 presumptive enterococci isolates representing one isolate from each steer fecal sample were revived on the same media from which they were initially isolated (BEA, BEA^E^ or BEA^T^; BD). Cultures were grown over 36 h at 37°C and two colonies were selected and suspended in 75 μL of TE (10 mM Tris, 1 mM EDTA, pH 8.0). Samples were heat lysed for 5 min using a thermomixer set at 98°C with shaking at 1000 RPM, followed by centrifugation at 10,000 × g for 5 min. The supernatant containing the genomic DNA was used as a source of template for all PCR reactions. Simultaneously, a subset of presumptive enterococci consisting of ~50% isolates of each category including treatment type, media type and sampling day were randomly selected for species identification. In this manner, 519 presumptive enterococci isolates were selected (Table 1). All of the 1029 isolates were screened by PCR with *Enterococcus* specific *groES-EL* primers Ent-ES-211-233-F and Ent-EL-74-95-R (Zaheer et al., 2012) for confirmation as *Enterococcus* spp. whereas the 519 selected isolates for species identification were further processed for sequencing of the *groES-EL* PCR product. Occasionally, the sequence results of the *groES-EL* PCR product varied from publically available databases. In order to characterize those *Enterococcus* spp. isolates correctly, multilocus sequencing including 16S rRNA, *atpA, pheS*, and *rpoA* genes was used to identify species. Detailed methodology can be found in the supplementary information (Supplementary Figure 1 and Supplementary Table 1). In cases where an isolate did not generate the *groES-EL* PCR product, i.e., was not an *Enterococcus* spp., PCR amplification and sequencing of the 16S rRNA gene using primers 27F (5′-AGAGTTTGATCMTGGCTCAG-3′) and 1492R (5′-TACGGYTACCTTGTTACGACTT-3′) was conducted for taxonomic identification.

**Table 1:** **Distribution of isolates characterized in this study**.

**Treatment^a^**	**Media used for selection^b^**	**Sampling day^c^**					**Total**
		**0**	**14**	**84**	**113**	**225**	
CON	BEA	24	20	25	25	23	119
	BEA^E^	6	8	17	16	9	58
	BEA^T^	9	9	19	22	19	79
T11	BEA	24	20	25	25	25	122
	BEA^E^	6	8	15	20	14	65
	BEA^T^	8	7	24	25	22	86
Total		77	72	125	133	112	519

a *Steers fed no antibiotics (control, CON) or tylosin phosphate (11 ppm; T11); administered continuously and withdrawn on day 197*.

b *Isolates were streaked onto bile esculin azide agar (BEA) containing no antibiotics, or amended with erythromycin (8 μg/mL; BEA^E^) or with tylosin (32 μg/mL; BEA^T^)*.

c *Sampling days began at day 0 (arrival at feedlot) prior to antibiotic administration and continued until the end of the feeding trial; sample day 0 and 14 were during the silage-based diet, day 84 during the transition diet and day 113 and 225 during the grain-based diet*.

A subset of 171 isolates representing major species (~25% coverage) and all minor species were subject to antimicrobial susceptibility testing. These selected isolates were subject to PCR-based identification of resistance determinants and PFGE profiling.

### Antimicrobial susceptibility testing

Disk susceptibility tests were conducted on 171 characterized enterococci isolates according to the CLSI documents M02-A11 and M100-S24 (Clinical and Laboratory Standards Institute, 2014a,b). The antimicrobials tested, suppliers and resistance breakpoints applied are listed in Table 2. Reference strains *Staphylococcus aureus* ATCC 25923® and *E. faecalis* ATCC 29212® were used as quality controls. Resulting zones of inhibition were read using the BioMic V3 imaging system (Giles Scientific, Inc., Santa Barbara, CA, USA) and classified as sensitive or resistant based on CLSI interpretive criteria (Clinical and Laboratory Standards Institute, 2014b), except for tigecycline which used EUCAST interpretive criteria (The European Committee on Antimicrobial Susceptibility Testing, (EUCAST), 2014). Neither EUCAST nor CLSI defined breakpoints exist for enterococci with tylosin, however the quality control range of tylosin disks (30 μg) has recently been acknowledged for *S. aureus* ATCC 25923® (Buß et al., 2014). Tylosin minimum inhibitory concentration (MIC) were established for a sub-set of isolates containing *erm*(B) or *msr*C, both genes or neither gene according to CLSI documents M100-S24 and M07-A9, with results reported in the supplementary information (Supplementary Figure 2). Isolates exhibiting a high MIC (≥128 μg/mL) to tylosin also contained the resistance determinant *erm*(B). Therefore, isolates harboring the resistance determinant *erm*(B) were given the designation of resistant to tylosin.

**Table 2:** **Antibiotics, suppliers, disk content and breakpoints used for disk susceptibility testing**.

**Antibiotic**	**Supplier**	**Disk content (μg)**	**Zone diameter (mm) breakpoints^d^**		
			***S***	***I***	***R***
Ampicillin^a^	BD	10	≥17	–	≤16
Doxycycline^a^	BD	30	≥16	13–15	≤12
Erythromycin^a^	BD	15	≥23	14–22	≤13
Gentamicin^a^	BD	120	≥10	7–9	6
Levofloxacin^a^	BD	5	≥17	14–16	≤13
Linezolid^a^	BD	30	≥23	21–22	≤20
Nitrofurantoin^a^	BD	300	≥17	15–16	≤14
Quinupristin-dalfopristin^a^	BD	4.5/10.5	≥19	16–18	≤15
Streptomycin^a^	BD	300	≥10	7–9	6
Tigecycline	BD	15	≥18	–	<15
Tylosin^b^	Medox	30	n/a	n/a	n/a
Vancomycin^a^^,^ ^c^	BD	30	≥17	15–16	≤14

a *M100-S24: Performance standards for antimicrobial susceptibility testing; twenty-fourth informational supplement (Clinical and Laboratory Standards Institute, 2014b)*.

b *Breakpoint tables for interpretation of MICs and zone diameters. Version 4.0. (The European Committee on Antimicrobial Susceptibility Testing, (EUCAST), 2014)*.

c *Vancomycin requires 24 h incubation while for all other antibiotics 16–18 h incubation is sufficient*.

d *Zone diameter value used to indicate susceptible (S), intermediate (I), resistant (R) and not available (n/a)*.

### Identification of resistance determinants

Of selected isolates, 125 isolates displaying intermediate or complete resistance to erythromycin were screened for the presence of macrolide resistance determinants. Isolates were first screened by PCR for the commonly found macrolide resistance determinants in enterococci, *erm*(B) and *msr*C (Portillo et al., 2000). For *erm*(B), PCR primers and reaction conditions were used as described by Chen et al. (2007). For *msr*C PCR, the forward and reverse primers, msrC_F1 (5′-TCGTTTTGTCATGAGACAAACAG-3′) and msrC_R1 (5′-AAATTAGTCGGTTCATCTAACAG-3′), respectively were used. A 20 μL PCR reaction using 2 μL of template DNA was prepared with the following reaction conditions: initial denaturation for 5 min at 95°C, followed by 35 cycles of denaturation for 30 s at 94°C, annealing for 30 s at 53°C, extension for 30 s at 72°C with a final extension for 10 min at 72°C. The PCR reaction product (5 μL) was resolved on a 2% agarose gel, and visualized for the presence of a 191 bp PCR product. An environmental sample, showing positive amplification for *msr*C and verified by DNA sequencing, was used as a positive control.

A subset of 40 isolates containing *erm*(B) or *msr*C or both genes and consisting of all identified species with a variety of PFGE profiles were further screened for the presence of other macrolide resistance determinants. These included *erm*(A), *erm*(C), *erm*(F), and *erm*(T) with primers and reaction conditions as described by Chen et al. (2007).

Isolates displaying intermediate or complete resistance to doxycycline were further screened for the tetracycline resistance determinants *tet*(B), *tet*(C), *tet*(L), and *tet*(M). A 20 μL PCR reaction using 2 μL of template DNA was prepared with products resolved on a 2% agarose gel. For *tet*(B), primers as described by Peak et al. (2007) were used with the following reaction conditions; initial denaturation for 5 min at 95°C, followed by 35 cycles of denaturation for 30 s at 94°C, annealing for 30 s at 60°C, extension for 30 s at 72°C, and a final extension for 10 min at 72°C. Primers and reaction conditions for *tet*(C), *tet*(L), and *tet*(M) were as described by Ng et al. (2001). The expected product size for *tet*(B), *tet*(C), *tet*(L), and *tet*(M) were 205, 418, 267, and 406 bp, respectively.

For all PCR reactions, the commercially available HotStarTaq Plus Master Mix Kit (Qiagen Canada, Inc., Mississauga, ON, Canada) was used according to manufacturer's instructions. Plasmids containing the corresponding gene fragments were used as positive controls (Alexander et al., 2009; Zaheer et al., 2013).

### PFGE

One-hundred and seventy-one isolates were subjected to PFGE profiling with *Sma*I restriction enzyme using a modified procedure of PulseNet USA (Center for Disease Control and Prevention, 2012). Briefly, bacteria grown overnight on brain-heart infusion-agar (BHI-agar; BD) were harvested using sterile swabs and suspended in TE buffer to an OD of 1.85 at 610 nm. An aliquot (400 μL) of cell suspension was transferred to a 1.5 mL microfuge tube containing 20 μL of lysozyme (50 mg/mL; Sigma-Aldrich, Co., St Louis, Mo, USA), gently mixed and incubated at 55°C for 45 min. An equal volume of 1.2% molten SeaKem Gold agarose (Lornza, Rockland, Maine, USA) in TE buffer was added and the mixture dispensed in duplicate into re-useable plug molds (Bio-Rad Laboratories, Hercules, CA, USA) and allowed to solidify at room temperature. Duplicate plugs were added to 2 mL microfuge tubes containing 1.8 mL cell lysis buffer [50 mM Tris; 50 mM EDTA; 1% sodium sarcosyl] and 9 μL of Proteinase K (20 mg/mL; Sigma-Aldrich, Co., St Louis, Mo, USA) and incubated for 2 h at 55°C with agitation (300 rpm). Plugs were washed twice in sterile, deionized H_2_O (1.8 mL) and three times in TE (1.8 mL) for 10 min each using a thermomixer set at 50°C and 300 rpm. Restriction digestion and electrophoresis conditions were as described by Zaheer et al. (2013). Gels were photographed using an AlphaImager gel documentation system (Alpha Innotech Corp., St. Leandro, CA, USA) and banding patterns analyzed with BioNumerics V6.6 software (Applied Maths Inc., Austin, TX, USA), using Dice coefficient and the unweighted pair group method (UPGMA). Optimization and band tolerance were both set at 1%. *Salmonella* serotype Braenderup digested with XbaI was included in each gel as a control reference and for normalization of band fragments.

### Data and statistical analysis

Enumeration data were used to determine the proportion of steers positive for macrolide resistant enterococci and the proportion of macrolide resistant *Enterococcus* in the total population. For the purposes of enumeration, esculin hydrolyzing colonies observed on BEA, BEA^E^, and BEA^T^ plates were assumed to be enterococci.

Data were analyzed using commercially available statistical analysis software (SAS Systems for Windows, version 9.3, SAS Institute Inc, Cary, NC, USA). Prior to analysis, enumeration data were normalized through a log transformation. When enumeration data for the antibiotic selective media exceeded that of the non-selective media for each sampling point, it was assumed that 100% of the population was resistant to the respective antibiotic. The MIXED procedure of SAS was used to assess CFU counts over time and the proportion of macrolide resistant enterococci in the total population. The CFU counts over time were analyzed with media type, day and media type × day in the model as fixed effects while for the proportion of macrolide resistant enterococci in the total population, day, treatment and day × treatment interaction were included in the model as fixed effects. For both analyses, day was included as a repeated measure. Results were considered significant when *P* < 0.05. For most sampling days, 50 samples were collected, but due to conflicts with other experiments in the feedlot facility, only 30 samples were collected on day 49, 141, 169, and 197.

## Results

### Prevalence of positive steers and CFU counts of macrolide resistant enterococci

**Figure 2:**
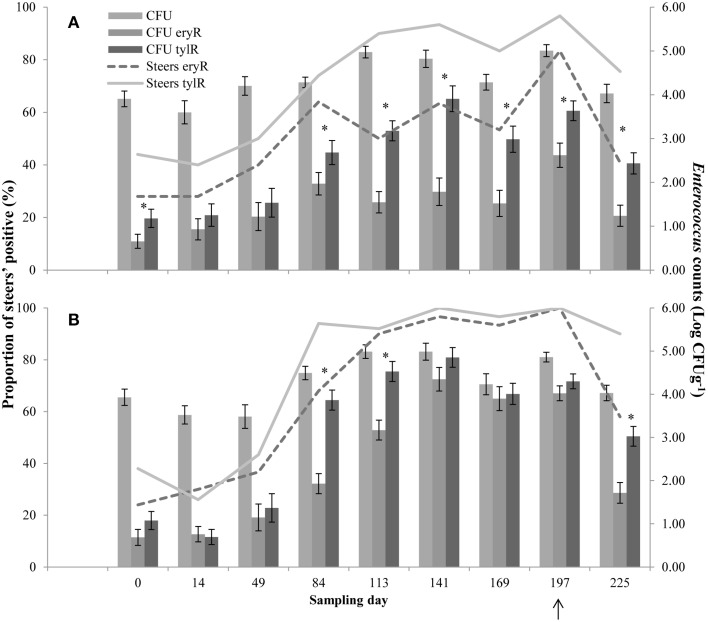
**Proportion of steers positive for ery^R^ enterococci (Steers eryR) or tyl^R^ enterococci (Steers tylR) and *Enterococcus* counts (log CFUg^−1^) of, total population (CFU), ery^R^ enterococci (CFU eryR) or tyl^R^ enterococci (CFU tylR) for CON (A) or T11 (B) treatments**. Arrow indicates when antibiotics were withdrawn from the diet. An “^*^” indicates days for which there was a significant difference between ery^R^ and tyl^R^ *Enterococcus* counts (*P* < 0.05). For each treatment (day 0, 14, 84, 113, and 225 *n* = 50; day 49, 141, 169, and 197 *n* = 30).

Upon arrival at the feedlot, 28 and 24% (CON and T11, respectively) of the steers were positive for ery^R^ enterococci, whilst 44 and 38% (CON and T11, respectively) were positive for tyl^R^ enterococci, even though steers did not previously receive antibiotics (Figure 2).

For the control group, the counts of tyl^R^ enterococci were higher (*P* < 0.05) than the counts of ery^R^ enterococci on d 0, 84, 113, 141, 169, 197, and 225 (Figure 2A). Whilst for the tylosin treatment, the counts of tyl^R^ enterococci were higher (*P* < 0.05) than the counts of ery^R^ enterococci for d 84, 113, and 225 (Figure 2B). In general, the counts of ery^R^ enterococci in the tylosin treatment group and counts of tyl^R^ enterococci in both treatment groups increased over the sampling period as the cattle were transitioned from a silage-based growing diet to a grain-based finishing diet (Figure 2). The increased counts of macrolide resistant enterococci over the experiment were due to an increase in the proportion of macrolide resistant enterococci within the total population.

### Proportion of macrolide resistant enterococci in the total enterococci population

**Figure 3:**
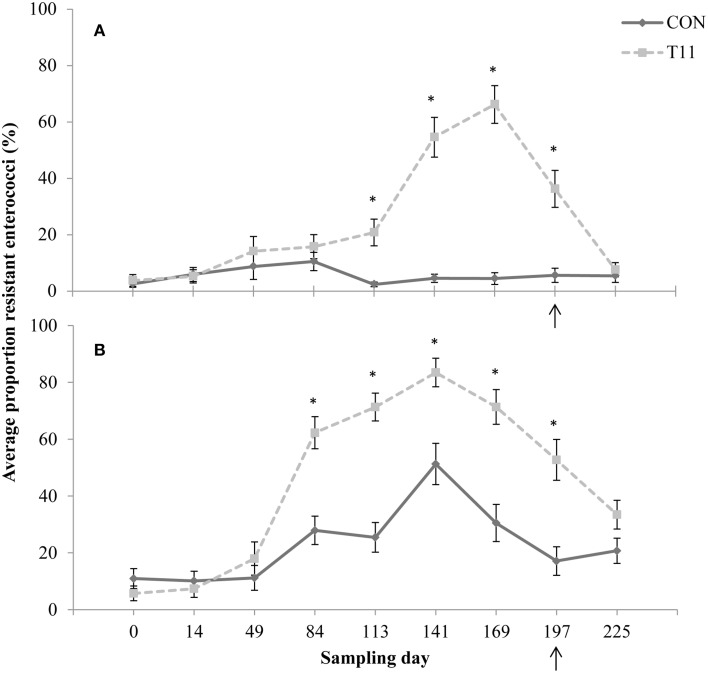
**Proportion of erythromycin-resistant (A) or tylosin-resistant (B) fecal enterococci isolates for both treatments across all sampling days**. Arrow indicates when antibiotics were withdrawn from the diet. Line styles distinguish the treatment. An “^*^” indicates days for which there was a significant difference between treatments (*P* < 0.05). For each treatment (day 0, 14, 84, 113, and 225 *n* = 50; day 49, 141, 169, and 197 *n* = 30).

No difference (*P* > 0.05) was observed between control and tylosin-fed steers on d 0, 14, 49, and 84 for the proportion of ery^R^ enterococci or d 0, 14, and 49 for the proportion of tyl^R^ enterococci (Figures 3A,B, respectively). On d 113, 141, 169, and 197, the proportion of ery^R^ enterococci was higher (*P* < 0.001) for steers fed tylosin compared to controls. The proportion of tyl^R^ enterococci, resistance was higher (*P* < 0.001) for steers fed tylosin compared to controls on d 84, 113, 141, 169, and 197. After withdrawal of tylosin on d 197, the proportion of ery^R^ or tyl^R^ enterococci decreased until there was no difference (*P* > 0.05) between tylosin-fed and control steers on d 225 (Figures 3A,B, respectively).

### Characterization of enterococci

**Figure 4:**
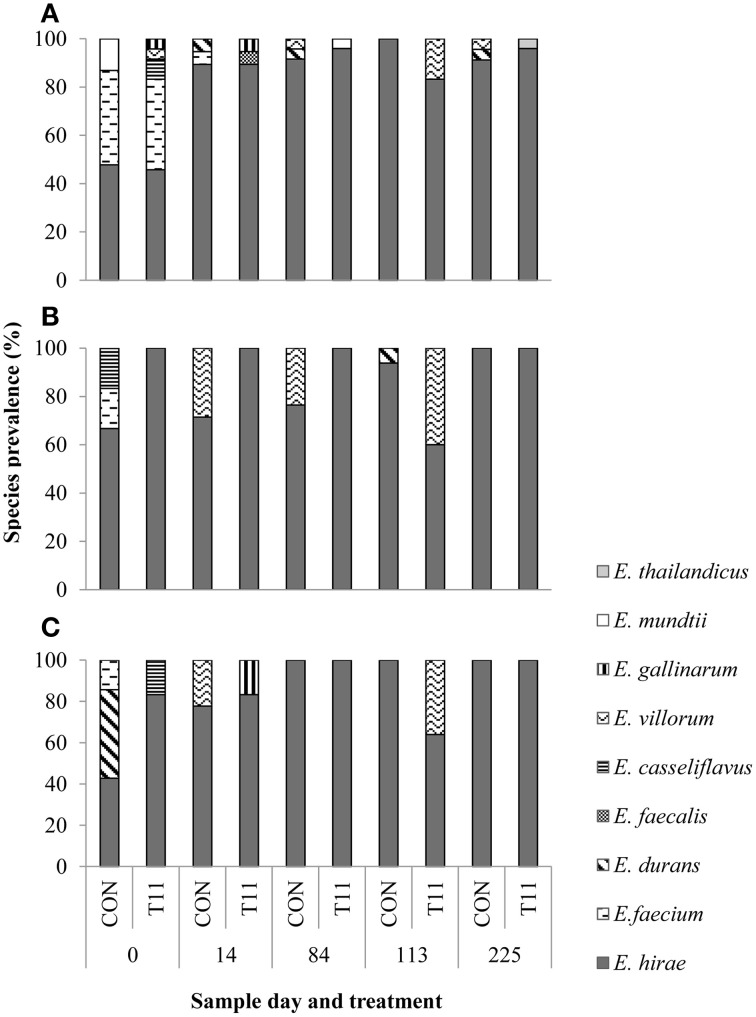
**Species distribution of characterized isolates from (A) BEA (bile esculin azide agar), (B) BEA^E^ (bile esculin azide agar amended with erythromycin [8 μg/mL]) and (C) BEA^T^ (bile esculin azide agar amended with tylosin [32 μg/mL])**. Prevalence was calculated by dividing the number of isolates for each species by the total number of isolates from each sample day and treatment.

Of the 1029 isolates analyzed, 95.2% were confirmed as enterococci by PCR. Of the 519 isolates speciated, 504 were identified as *E. hirae* (*n* = 431), *Enterococcus villorum* (*n* = 32), *E. faecium* (*n* = 21), *Enterococcus durans* (n = 7), *Enterococcus casseliflavus* (*n* = 4), *Enterococcus mundtii* (*n* = 4), *Enterococcus gallinarum* (*n* = 3), *E. faecalis* (*n* = 1), and *Enterococcus thailandicus* (*n* = 1). The remaining 15 non-enterococci were identified as *Lactobacillus* spp. (*n* = 3), *Aerococcus* spp. (*n* = 9), *Streptococcus* spp. (*n* = 2), and *Staphylococcus epidermids* (*n* = 1) as determined by 16S rRNA sequencing. All the species identified were represented by the 231 isolates originally recovered from BEA, whereas only six species (*E. hirae, E. villorum, E. faecium, E. durans, E. casseliflavus*, and *E. gallinarum*) were isolated from BEA^E^ and BEA^T^ (Figure 4). Variants of the *groES-EL* sequence for two isolates of *E. faecium* and single isolates of *E. thailandicus* and *E. villorum* have been submitted to the NCBI database (Accession numbers KP993544, KP993545, KP993546, and KP993547, respectively). The diversity of enterococci tended to be greater in steers upon arrival than at exit from the feedlot. A greater diversity of enterococci species were isolated from non-selective BEA compared with either BEA^E^ or BEA^T^, with similar proportions of most species occurring in control and tylosin-fed steers. *E. hirae* was the predominant species isolated from both control and tylosin-fed steers across all sampling dates (Figure 4).

### Antibiotic susceptibility testing

A subset (*n* = 171) of enterococci representing all of the isolated *Enterococcus* species were tested for antibiotic susceptibility (Table 3). Resistance to ampicillin, gentamicin, linezolid, streptomycin or tigecycline was not detected in any of the isolates. Vancomycin resistance was also absent in all isolates except for one which displayed intermediate resistance. One isolate of *E. casseliflavus* exhibited ERY-TYL-Q-D-van resistance and one isolate of *E. durans* exhibited ERY-TYL-q-d (lower case denotes intermediate resistance and upper case complete resistance). One isolate of *E. faecium* was ERY-DOX-TYL-q-d resistant, with other single isolates exhibiting intermediate ery-nit, ery-lvx or dox-nit-lvx-q-d resistance. Two isolates of *E. gallinarum* showed ery-TYL resistance and a number of *E. hirae* isolates were resistant to ERY-TYL (*n* = 27), ery-TYL (*n* = 27), ERY-dox-TYL (*n* = 8), or ERY-TYL-q-d (*n* = 7). With one exception, all *E. villorum* isolates exhibited ERY-TYL (*n* = 31) resistance.

**Table 3:** **Number of enterococci isolates (percentage of total species^a^) showing intermediate or complete resistance to antibiotics pooled across treatments, isolation media and sample date**.

**Enterococcus spp**.		**Antibiotic^b^ (No. isolates [%])**											
		**AMP**	**DOX**	**ERY**	**GEN**	**LVX**	**LZD**	**NIT**	**Q-D**	**STR**	**TGC**	**TYL^c^**	**VAN**
*E. hirae* (*n* = 98)	I	n/a	8 (8.2)	27 (27.6)	0	0	0	0	7 (7.1)	0	n/a	n/a	0
	R	0	0	42 (42.9)	0	0	0	0	0	0	0	69 (70.4)	0
*E. villorum*(*n* = 32)	I	n/a	0	0	0	0	0	0	0	0	n/a	n/a	0
	R	0	0	31 (96.9)	0	0	0	0	0	0	0	31 (96.9)	0
*E. faecium* (*n* = 21)	I	n/a	1 (4.8)	5 (23.8)	0	2 (9.5)	0	2 (9.5)	2 (9.5)	0	n/a	n/a	0
	R	0	1 (4.8)	14 (66.7)	0	0	0	0	0	0	0	1 (4.8)	0
*E. durans* (*n* = 7)	I	n/a	0	0	0	0	0	0	1 (14.3)	0	n/a	n/a	0
	R	0	0	1 (14.3)	0	0	0	0	0	0	0	1 (14.3)	0
*E. casseliflavus* (*n* = 4)	I	n/a	0	0	0	0	0	0	0	0	n/a	n/a	1 (25.0)
	R	0	0	2 (50.0)	0	0	0	0	1 (25.0)	0	0	2 (50.0)	0
*E. mundtii (n = 4)*	I	n/a	0	0	0	0	0	0	0	0	n/a	n/a	0
	R	0	0	0	0	0	0	0	0	0	0	0	0
*E. gallinarum (n = 3)*	I	n/a	0	2 (66.7)	0	0	0	0	0	0	n/a	n/a	0
	R	0	0	0	0	0	0	0	0	0	0	2 (66.7)	0
*E. faecalis* (*n* = 1)	I	n/a	0	0	0	0	0	0	0	0	n/a	n/a	0
	R	0	0	0	0	0	0	0	1 (100.0)	0	0	0	0
*E. thailandicus* (*n* = 1)	I	n/a	0	1 (100.0)	0	0	0	0	0	0	n/a	n/a	0
	R	0	0	0	0	0	0	0	0	0	0	0	0

a *Percentages were calculated by dividing resistant isolates with the total number of isolates for individual species and rounded to the first decimal place*.

b *AMP, ampicillin; DOX, doxycycline; ERY, erythromycin; GEN, gentamicin; LVX, levofloxacin; LZD, linezolid; NIT, nitrofurantoin; Q-D, quinupristin-dalfopristin; STR, streptomycin; TGC, tigecycline; TYL, tylosin; VAN, vancomycin*.

c *Resistance isolates were classified as those which carried the erm(B) resistance gene (see Section Materials and Methods for more information)*.

*R, complete resistance; I, intermediate resistance; n/a, no interpretive criteria for intermediate resistance*.

In general, isolates grown on BEA^T^ also exhibited erythromycin resistance. An exception to this was three isolates of *E. durans* isolated on BEA^T^, which remained susceptible to erythromycin.

### Identification of resistance determinants

Of the 125 enterococci isolates displaying intermediate or complete resistance to erythromycin, the *erm*(B) gene was detected in 106 isolates representing *E. hirae*, *E. durans*, *E. faecium, E. villorum, E. gallinarum*, and *E. casseliflavus*. Of the 19 erythromycin-resistant *E. faecium* isolates obtained all except one lacked *erm*(B), but all were positive for *msr*C. The isolate identified as *E. thailandicus* displayed intermediate resistance to erythromycin, but was negative for all of the macrolide resistance determinants tested. None of the isolates tested positive for the other macrolide resistance determinants.

A total of 10 isolates displayed intermediate or complete resistance to doxycycline. None of the isolates were positive for *tet*(B) or *tet*(C). All 10 isolates were positive for *tet(*M) and 9 were positive for *tet*(L).

### PFGE

**Figure 5:**
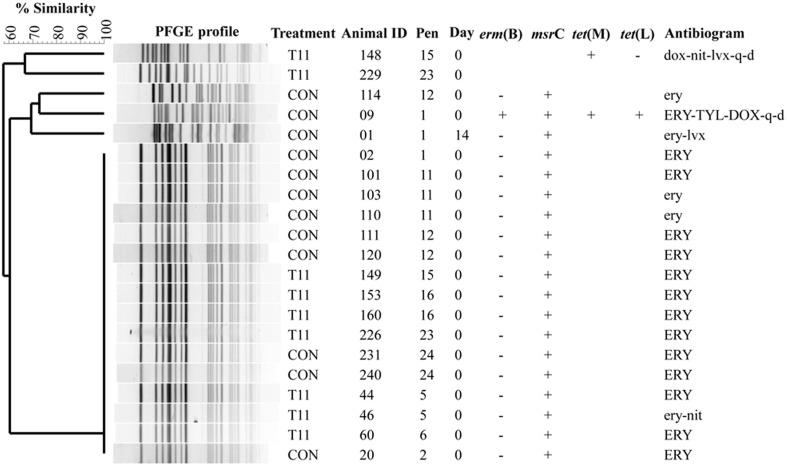
**Dendrogram of PFGE *Sma*I profiles from isolates identified as *Enterococcus faecium***. A “+” indicates PCR positive and “−” indicates PCR negative to the respective genes. A “blank” space indicates the gene was not screened for in the respective isolate. For the antibiogram, upper case denotes complete resistance and lower case denotes incomplete resistance.

**Figure 6:**
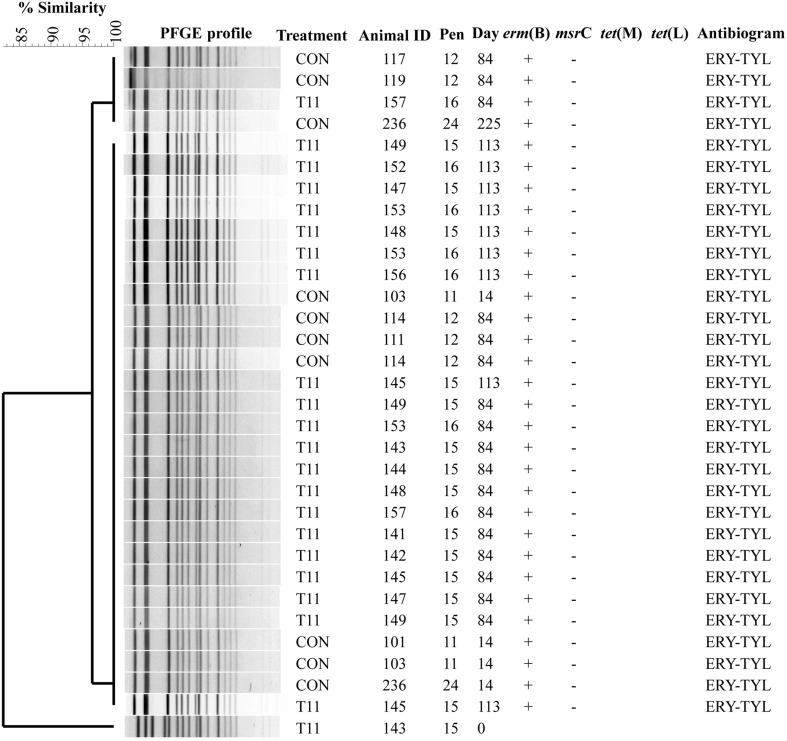
**Dendrogram of PFGE *Sma*I profiles from isolates identified as *Enterococcus villorum***. A “+” indicates PCR positive and “−” indicates PCR negative to the respective genes. A “blank” space indicates the gene was not screened for in the respective isolate. For the antibiogram, upper case denotes complete resistance and lower case denotes incomplete resistance.

**Figure 7:**
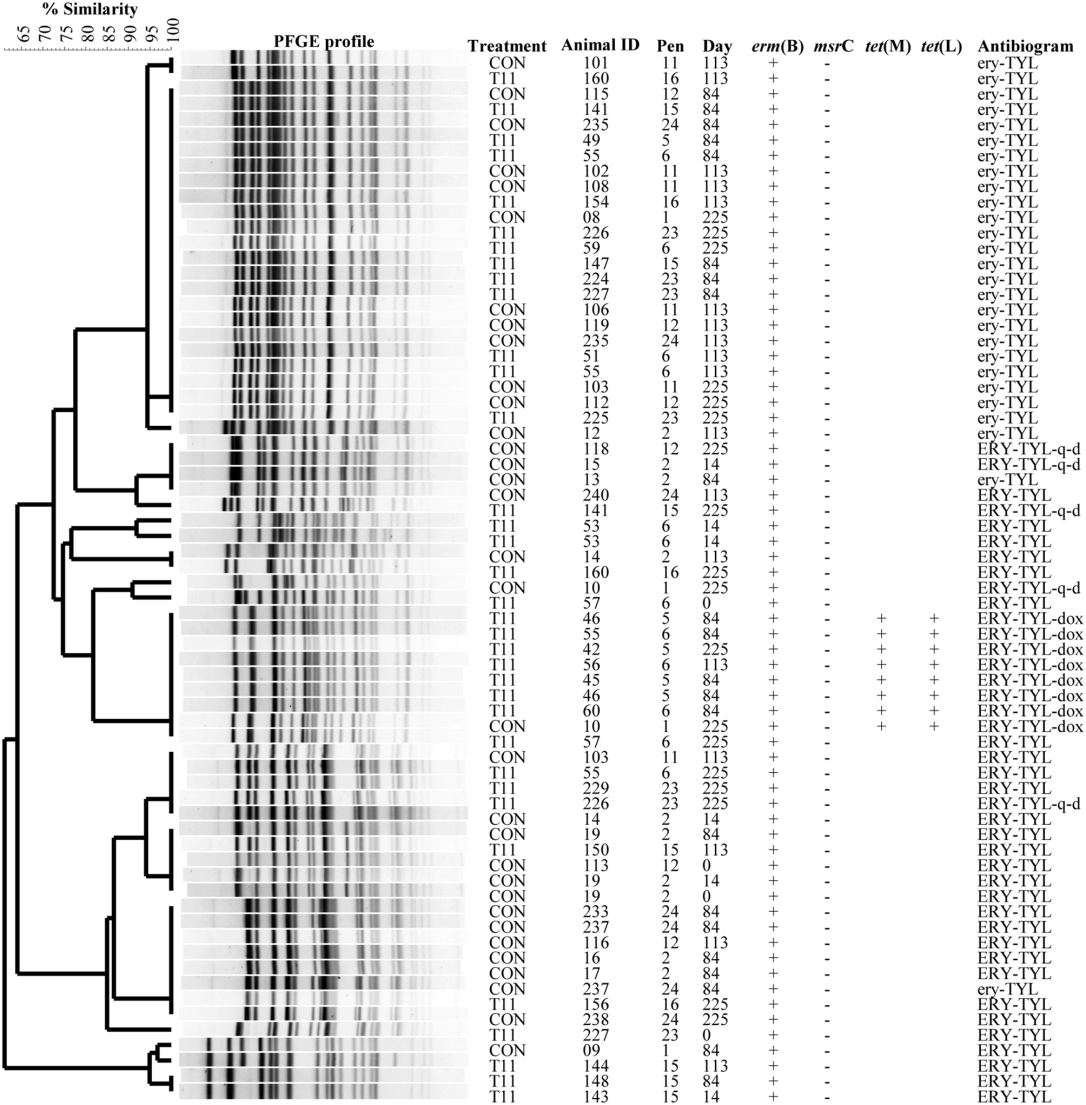
**Dendrogram of PFGE *Sma*I profiles from isolates identified as erythromycin resistant *Enterococcus hirae***. A “+” indicates PCR positive and “−”indicates PCR negative to the respective genes. A “blank” space indicates the gene was not screened for in the respective isolate. For the antibiogram, upper case denotes complete resistance and lower case denotes incomplete resistance.

The PFGE profiles of *E. faecium, E. villorum* and erythromycin resistant *E. hirae* are displayed in Figures 5–7, respectively. *E. faecium* had at least 16 isolates from different steers with the same PFGE profile, suggesting the presence of a clonal population. Isolates from this clonal population were isolated only on day 0 (Figure 5). The similarity (>95%) of PFGE profiles of *E. villorum* also suggested clonality (Figure 6). Unlike *E. faecium*, these profiles appeared on day 14 of the trial and persisted until the end of the experiment. PFGE profiles of erythromycin resistant *E. hirae* produced 8 clusters with >85% similarity (Figure 7).

## Discussion

Enterococci are ubiquitous in nature and are frequently isolated from the gastrointestinal tract of mammals, including humans (Franz et al., 2011). Of the enterococci recovered from this study *E. hirae* was revealed to be the predominant species isolated, an observation consistent with previous studies (Anderson et al., 2008; Jackson et al., 2010; Zaheer et al., 2013).

Enterococci have been described as a “drug resistance gene trafficker” due to the ease with which they can acquire and transfer resistance genes (Werner et al., 2013). They have emerged as a serious threat to human health, particularly due to the acquisition of vancomycin resistance, increasing the difficulty of successful treatment (Center for Disease Control and Prevention, 2013). Of the 171 isolates examined for antibiotic resistance, only one isolate displayed intermediate resistance to vancomycin. This isolate was identified as *E. cassiflavus*, an outcome that likely reflects the intrinsic resistance of *E. casseliflavus* and *E. gallinarum* to low levels of vancomycin (Hollenbeck and Rice, 2012). This observation is encouraging, as the enterococci isolated from beef cattle do not appear to represent a significant source of vancomycin resistance.

*E*. *faecium* and *E. faecalis* are the two species most commonly associated with nosocomial human infections (Ruoff et al., 1990; Werner et al., 2008; Sievert et al., 2013). These species have been isolated from cattle (Kuhn et al., 2003; Anderson et al., 2008; Jackson et al., 2010), but they do not predominate, with our study suggesting that their prevalence declines after cattle enter the feedlot. Although *E. hirae*, as well as other enterococcal species (i.e., *Enterococcus avium, E. durans, E. casseliflavus, E. gallinarum*, and *Enterococcus raffinosus*) can cause clinical infections in humans, they are rare and thought to be more opportunistic in nature than those caused by *E*. *faecium* and *E. faecalis* (Ruoff et al., 1990; Alfouzan et al., 2014). Presence of *E. hirae* predominantly in the bovine gastrointestinal tract suggests that cattle do not present a significant source of *Enterococcus* that could colonize and infect humans.

In the absence of selection, the predominant resistance phenotype observed in the enterococci recovered from cattle was to erythromycin or tylosin, including isolates recovered pre- and post- antibiotic treatment. Despite no prior treatment with antimicrobials, steers harbored ery^R^ (28 and 24%, CON and T11 respectively) and tyl^R^ (44 and 38%, CON and T11 respectively) enterococci upon arrival at the feedlot (Figure 2). This suggests that naturally occurring resistance determinants coding for macrolide resistance are already present and circulating in bovine gut enterococci populations.

For some days, the counts of tyl^R^ enterococci were higher (*P* < 0.05) than ery^R^ enterococci for both treatment groups (Figure 2). It would be expected that similar counts would be obtained for both ery^R^ and tyl^R^ enterococci as the same resistance mechanism confers resistance to both antibiotics (Roberts, 2008; Desmolaize et al., 2011). Enterococci with both intermediate and complete resistance to erythromycin were isolated from tylosin plates; erythromycin plates only selected for enterococci with complete resistance to erythromycin, explaining some of the discrepancy seen between enumeration data for the two media. Isolates from tylosin media with intermediate resistance to erythromycin also carried the *erm*(B) gene. It appears that the MIC breakpoint for erythromycin may be too high, therefore missing enterococci with intermediate resistance which also carry a resistance determinant. Conversely, the MIC breakpoint for tylosin may be too low thereby selecting for isolates that contain resistance determinants that may be compromised, resulting in an intermediate resistance phenotype. The fact that three isolates of *E. durans* from the tylosin media remained susceptible to erythromycin supports this theory. It is possible however, that these isolates carry a resistance determinant not screened for. It would be worthwhile to further explore the likely genetic differences between the resistance determinant(s) from complete and intermediate tylosin resistant isolates to identify the linkage between antimicrobial resistance (AMR) genotype and phenotype.

As the trial progressed, the number of steers positive for macrolide resistant enterococci increased in both treatment groups. This increase, even in the control group may be a reflection of increased transmission between steers due to close proximity in the feedlot environment. Likewise, the changing population dynamics of enterococci in the gastrointestinal tract of cattle may also contribute to increased transmission. Increased shedding of macrolide resistant enterococci would increase the likelihood of cattle being exposed to macrolide resistant enterococci and thus also increase the detection of positive cattle. Similarly, an increase in the proportion of the population that are macrolide resistant would increase the chances of isolating macrolide resistant enterococci. For a steer to be considered positive in this study, isolation of a single macrolide resistant enterococci colony was required. In order to make an assessment of resistance development it is important to look at resistance as a proportion of the total enterococci population.

The CFU counts of the overall enterococci population remained relatively constant over the experiment for both treatments (Figure 2). This trend was also true for CFU counts of ery^R^ enterococci in the control group (Figure 2A), whilst the CFU counts of ery^R^ enterococci in the tylosin treatment group tended to increase during the period of tylosin administration before dropping off on d 197, presumably due to its withdrawal from the diet (Figure 2B). This trend was also observed for the CFU counts of tyl^R^ enterococci for both treatments, with possible differences between ery^R^ and tyl^R^ CFU being attributed to the selection of intermediate resistant enterococci on the tylosin media (Figure 2). A delay between the increase of CFU counts and tylosin administration can be seen, with increases coinciding with the transition from a silage-based diet to a grain-based diet.

High-grain diets tend to increase the amount of starch available in the lower intestinal tract, changing the nutrient availability for bacterial growth (Callaway et al., 2009). Previous researchers have reported a 1 (Scott et al., 2000) to 3 log (Diez-Gonzalez et al., 1998) increase in *Escherichia coli* when cattle were transitioned from a forage- to a grain-based diet. Changes that occur in the gastrointestinal environment of cattle as a result of increased starch in the diet alter the composition of the microbiome (Shanks et al., 2011). It is possible that the transition to a grain-based diet created conditions ideal for proliferation of macrolide resistant enterococci. Although not seen with the CFU of ery^R^ enterococci, the increase of tyl^R^ enterococci in both the control and tylosin treatment group, suggest factors other than administration of tylosin may have been selecting for macrolide resistant enterococci.

Increases in ery^R^ enterococci in cattle as a result of the administration of tylosin has been previously documented (Jacob et al., 2008; Zaheer et al., 2013), but these authors did not study the effect of withdrawal of tylosin from the diet. As in previous studies, there was an increase in the proportion of ery^R^ and tyl^R^ resistant enterococci isolated from cattle administered tylosin. The proportion of ery^R^ and tyl^R^ resistant enterococci for the tylosin treatment began decreasing just prior to removal of tylosin from the diet and continued to decrease after its withdrawal, until no difference (*P* > 0.05) was observed between treatments on d 225 (Figure 3). It appears that withdrawal of tylosin phosphate prior to slaughter contributes to a reduction in the proportion of macrolide resistant enterococci entering the food chain. However, the possibility that other unknown factors such as stress, age and diet may also be influencing this decline cannot be eliminated. It would be interesting to investigate this phenomenon further to determine why this reduction is occurring prior to the withdrawal of tylosin from the diet.

A decrease in *Enterococcus* species diversity was observed as the experiment progressed, with *E. hirae* being the predominant species identified. Transitioning of the diet from a forage- to a grain-based diet alters the fecal microbiome of cattle (Shanks et al., 2011). Diet may be a contributing factor in the shift in species diversity seen in this study, but it is also possible that other factors, such as age, may be influencing the fecal microbial community (Devriese et al., 1992).

In this study, *E. thaliandicus* and *E. villorum* were identified using multilocus sequencing of 16S rRNA, *atpA, pheS*, and *rpoA* genes after the discovery of *groES-EL* PCR products that varied from publically available databases (Supplementary Figure 1). To our knowledge, these species have not been previously isolated from cattle. *E. thailandicus* was first isolated in 2008 from fermented sausage in Thailand (Tanasupawat et al., 2008) and has been found in swine feces (Liu et al., 2013). *E. villorum* was first isolated in 2001 from piglets (Vancanneyt et al., 2001). Traditional methods of identifying *Enterococcus* species rely on biochemical tests which are unreliable for atypical species or species that have not been previously isolated (Deasy et al., 2000; Jackson et al., 2004). Molecular techniques have the advantage of being able to differentiate between closely related enterococci species.

Erythromycin resistant enterococci possessed either *erm*(B) or *msr*C or both resistance genes. Isolates designated as tylosin resistant possessed *erm*(B). Other macrolide resistance determinants were absent in the subset of isolates screened and it is possible that isolates not screened may have contained macrolide resistance determinants other than *erm*(B) or *msr*C. Presence of at least one resistance determinant in these isolates however confirmed the association between resistance phenotype and genotype.

Eight isolates of *E. hirae* and one isolate of *E. faecium* displayed complete resistance to erythromycin and either complete or intermediate resistance to doxycycline. These isolates were all positive for *erm*(B), *tet*(L), and *tet*(M). The resistance genes *erm*(B) and *tet*(M) are often associated with the transposon Tn*1545* (Clewell et al., 1995; Rice, 1998). The transposon integrase gene (*int* gene) of Tn*916*/Tn*1545* family of transposons has been previously detected in enterococci (De Leener et al., 2004). The identification of *erm*(B) and *tet*(M) in the same isolate in this study could possibly suggest the presence of mobile genetic elements. It would be worthwhile to investigate this further as many *erm* genes are often linked with other antibiotic resistance genes, tetracycline in particular (Roberts et al., 1999). Linkage of macrolide and other resistance genes is potentially problematic as administrating tylosin to cattle may not only select for macrolide resistance, but also for resistance to antibiotics such as tetracycline. Co-selection of tetracycline resistance upon the administration of tylosin has been suggested to occur within the fecal microbial communities of beef cattle (Chen et al., 2008). Linkage of these genes on mobile genetic elements increases the potential for the transfer of genes conferring resistance to multiple antibiotics (Hegstad et al., 2010; Tremblay et al., 2012).

Pulsed-field gel electrophoresis revealed a predominate cluster of *E. faecium* containing *msr*C and displaying a similar AMR profile of intermediate or complete resistance to erythromycin. Sequencing of *msr*C revealed that all isolates within this cluster had identical sequences. However, there were sequence differences in the *msr*C gene among these isolates and isolates with unique PFGE profiles (Figure 5). The four newly identified sequences have been submitted to the NCBI sequence database (Accession numbers KP775623, KP775624, KP775625, and KP775626).

Similar PFGE profiles were seen pre- and post-antibiotic treatment for erythromycin resistant *E. hirae*, highlighting that administration of tylosin selected for erythromycin resistant enterococci already present in the bovine gastrointestinal tract. These same profiles were still present after d 225; 28 days after tylosin had been removed from the diet. This suggests that although administration of tylosin increased the proportion of macrolide resistant enterococci in beef cattle it does not appear to be promoting the transfer of resistance between isolates. Once the selection pressure is removed (withdrawal of tylosin), the proportion of macrolide resistant enterococci returned to levels seen before antibiotic treatment.

## Conclusion

Few studies have investigated the role that administration of tylosin in the feed of beef cattle has on the development of macrolide resistance in enterococci. This study demonstrated that administering tylosin to cattle increases the proportion of macrolide resistant enterococci. Withdrawal of tylosin from the diet appears to contribute to the decline in macrolide resistant enterococci but may not be the only factor influencing this decline. Furthermore, transitioning cattle to a grain based diet appears to alter the species population of enterococci to one in favor of *E. hirae*, a species not commonly associated with infection in humans. PFGE profiling of erythromycin resistant *E. hirae* suggest that antibiotic administration selects resistant strains already present in the intestinal microbial population.

### Conflict of interest statement

The authors declare that the research was conducted in the absence of any commercial or financial relationships that could be construed as a potential conflict of interest.
