# Supplementary material for: Effect of in-feed administration and withdrawal of tylosin phosphate on antibiotic resistance in enterococci isolated from feedlot steers
Source: Front Microbiol. 2015 May 27;6:483. doi: 10.3389/fmicb.2015.00483 (PMC4444845; doi:10.3389/fmicb.2015.00483)
Supplement: Supplementary file 1 [file DataSheet1.DOCX]

***Supplementary Material***

Alicia G. Beukers^1,2^, Rahat Zaheer^2^, Shaun R. Cook^2^, Kim Stanford^3^, Alexandre V. Chaves^1^, Michael P. Ward^1^ and Tim A. McAllister^2*^

*^1^Faculty of Veterinary Science, The University of Sydney, Sydney, NSW, Australia*

*^2^Lethbridge Research Centre, Agriculture and Agri-Food Canada, Lethbridge, AB, Canada*

*^3^Alberta Agriculture and Rural Development, Lethbridge Research Centre, Lethbridge, AB, Canada*

***Correspondence:** Dr. Tim A. McAllister, Lethbridge Research Centre, Agriculture and Agri-Food Canada, 5403-1^st^ Ave. South, Lethbridge, AB, T1J 4B1, Canada.

[tim.mcallister@agr.gc.ca](mailto:tim.mcallister@agr.gc.ca)

1. **Verification of species identity of isolates with unique *groES-EL* intergenic spacer regions**

Of the isolates examined for speciation, 36 of these presented unique *groES-EL* intergenic spacer regions not currently deposited in the NCBI database. Of these 36, 5 unique sequences were present (Supplementary Figure 1). Genetic methods using 16S rRNA, *atpA*, *pheS* and *rpoA* gene sequencing were used to further verify the identity of these *Enterococcus* species. The genes *atpA*, *pheS* and *rpoA* were selected as good candidates for *Enterococcus* species identification due to their high discriminatory power (Naser et al. 2005a; Naser et al. 2005b). The sequences of the primers used for amplification and sequencing of 16S rRNA, *atpA*, *pheS* and *rpoA* genes are listed in Supplementary Table 1. Different primer combinations were used to amplify *atpA*, *pheS* and *rpoA* based on the species each isolate was speculated to be following 16S rRNA amplification and sequence analysis. For *Enterococcus thailandicus,* the primer combinations atpA E. thai; rpoA specific and pheS specific were used, for *Enterococcus villorum,* atpA E. vill; rpoA specific and pheS specific were used, for *Enterococcus faecium,* atpA all; rpoA specific and pheS specific were used and for *Enterococcus casseliflavus* atpA E. cass, rpoA specific and pheS E. cass were used. These primers were designed using *atpA*, *pheS* and *rpoA* partial gene sequences of enterococci species publicly available. For each gene, a 50 μL reaction using 5 μL of DNA template was set up using a final primer concentration of 500 nM, except for atpA all and rpoA all where 1000 nM was used. PCR products were purified using commercial kits and sequenced using both the forward and reverse primers. The reaction conditions were as follows; initial denaturation for 5 min at 95°C, followed by either 35 or 40 cycles of denaturation for 30 s at 94°C, annealing at temperature specified in table for 30 s, extension at 72°C with time specified in table and with a final extension for 10 min at 72°C. Sequence results were BLAST against the non-redundant database to further confirm species identification.

1. **Supplementary Figures and Tables**
   1. **Supplementary Tables**

**Supplementary Table 1:** Primers for 16S rRNA, *atpA, pheS* and *rpoA* amplification and sequencing

| **Primer name** |  | **Primer sequence (5’-3’)** |  | **Expected product size (bp)** |  | **Annealing temperature (°C)** |  | **Number of cycles** |  | **Extension time** |  | **Reference** |
| --- | --- | --- | --- | --- | --- | --- | --- | --- | --- | --- | --- | --- |
| 27F |  | AGAGTTTGATCCTGGCTCAG |  | ~1400 |  | 58 |  | 35 |  | 1 m 30 s |  | Zaheer et al., 2013 |
| 1492R |  | GGTTACCTTGTTACGACTT |  |  |  |  |  |  |  |  |  |  |
|  |  |  |  |  |  |  |  |  |  |  |  |  |
| atpA all F |  | GGDYTWGAAAAYGCVATGAGTG |  | 1070 |  | 49 |  | 40 |  | 1 m |  | This study |
| atpA all R |  | CCRAAYTGNGTRAADGCTTC |  |  |  |  |  |  |  |  |  |  |
|  |  |  |  |  |  |  |  |  |  |  |  |  |
| atpA E. thai F |  | GAATGCATGAGTGGTGAGTTGC |  | 1054 |  | 59 |  | 35 |  | 1 m 30 s |  | This study |
| atpA E. thai R |  | GCGTAAATGCTTCAAGTTCACGG |  |  |  |  |  |  |  |  |  |  |
|  |  |  |  |  |  |  |  |  |  |  |  |  |
| atpA E. vill F |  | CGTGCACATGGGTTAGAAAACGC |  | 1098 |  | 59 |  | 35 |  | 1 m 30 s |  | This study |
| atpA E. vill R |  | TGTCGCAGCATCTAAATCAGAACC |  |  |  |  |  |  |  |  |  |  |
|  |  |  |  |  |  |  |  |  |  |  |  |  |
| atpA E. cass F |  | ACGGTTCTTATGGGATGGCA |  | 945 |  | 58 |  | 35 |  | 1 m |  | This study |
| atpA E. cass R |  | TCTGTGCAGAACCACCAACC |  |  |  |  |  |  |  |  |  |  |
|  |  |  |  |  |  |  |  |  |  |  |  |  |
| pheS specific F |  | CGDACVATGGAAAAACATG |  | 337 |  | 51 |  | 35 |  | 40 s |  | This study |
| pheS specific R |  | CWGCNCCTAARATYTCRATC |  |  |  |  |  |  |  |  |  |  |
|  |  |  |  |  |  |  |  |  |  |  |  |  |
| pheS E. cass F |  | GAAGTGCTGATTCGGACCCA |  | 415 |  | 58 |  | 35 |  | 40 s |  | This study |
| pheS E. cass R |  | CGATCCCTGACATTTCTAAGACG |  |  |  |  |  |  |  |  |  |  |
|  |  |  |  |  |  |  |  |  |  |  |  |  |
| rpoA all F |  | CGTCGTATYYTDYTDTCTTC |  | 485 |  | 48.9 |  | 40 |  | 40 s |  | This study |
| rpoA all R |  | CCRTCWGTCCADATYTCC |  |  |  |  |  |  |  |  |  |  |
|  |  |  |  |  |  |  |  |  |  |  |  |  |
| rpoA specific F |  | GGTGTACTGCATGAATTCTC |  | 548 |  | 54 |  | 35 |  | 40 s |  | This study |
| rpoA specific R |  | CTTTYTCVACCATGATTTCAGC |  |  |  |  |  |  |  |  |  |  |

**2.2 Supplementary Figures**


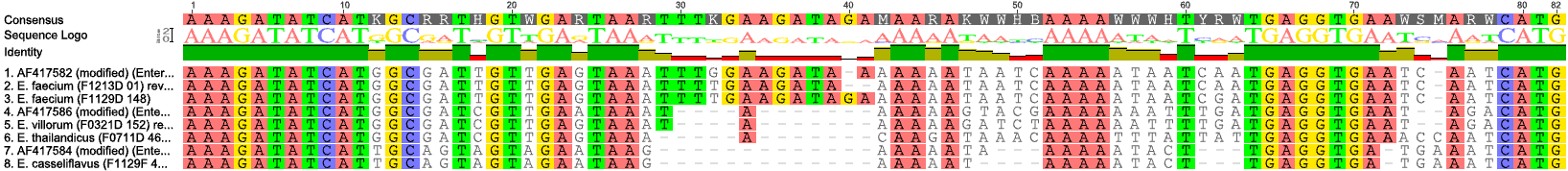


Consensus

Sequence Logo

Identity

ATCC 19434 *Enterococcus faecium*

*E. faecium* (F1213D 01)

*E. faecium** (F1129D 148)

ATCC 8043 *Enterococcus hirae*

*Enterococcus villorum* (F0321D 152)

*Enterococcus thailandicus* (F0711D 46)

ATCC 25788 *Enterococcus casseliflavus*

*Enterococcus casseliflavus* (F1129F 46)

**Supplementary Figure 1:** Alignment of *groES-EL* spacer region of *Enterococcus* species with unique spacer regions isolated from this study deposited in the NCBI database (Accession numbers KP993544, KP993545, KP993546 and KP993547). *Enterococcus casseliflavus* (F1129F 46) displays a ‘variant’ spacer region that has been reported previously (Tsai et al., 2005 and Zaheer et al., 2012). ATCC strains 19434, 8043 and 25788 were included as comparison. Stop codon of *groES* (TAA) and start codon of *groEL* (ATG) are underlined. Species were verified by sequencing and blast analysis of 16S rRNA, *atpA*, *pheS* and *rpoA*.

| **MIC (µg/mL)** | 128 | **1** | **1** |  |  | **1** |  | **3** |  |  |  |  |  |  |  |  |  |  |  |  |  |  |  |  |  |  |  |  |
| --- | --- | --- | --- | --- | --- | --- | --- | --- | --- | --- | --- | --- | --- | --- | --- | --- | --- | --- | --- | --- | --- | --- | --- | --- | --- | --- | --- | --- |
|  | 64 |  |  |  |  |  |  |  |  |  |  |  |  |  |  |  |  |  |  |  |  |  |  |  |  |  |  |  |
|  | 32 |  |  |  |  |  |  |  |  |  |  |  |  |  |  |  |  |  |  |  |  |  |  |  |  |  |  |  |
|  | 16 |  |  |  |  |  |  |  |  |  |  |  |  |  |  |  |  |  |  |  |  |  |  |  |  |  |  |  |
|  | 8 |  |  |  |  |  |  |  |  |  |  |  |  |  |  | 1 |  |  |  | 1 |  | 1 |  |  |  |  |  |  |
|  | 4 |  |  |  |  |  |  |  |  |  |  |  |  | 1 | 1 |  | 1 | 1 | 1 |  |  |  |  |  |  |  |  |  |
|  | 2 |  |  |  |  |  |  |  |  |  |  |  | 1 |  |  |  |  |  |  | 1 |  |  |  |  |  |  |  |  |
|  | 1 |  |  |  |  |  |  |  |  |  |  |  |  |  |  |  |  |  |  |  |  |  |  |  |  |  |  |  |
|  | 0.5 |  |  |  |  |  |  |  |  |  |  |  |  |  |  |  |  |  |  |  |  |  |  |  |  |  |  |  |
|  | 0.25 |  |  |  |  |  |  |  |  |  |  |  |  |  |  |  |  |  |  |  |  |  |  |  |  |  |  |  |
|  |  | <6 | 7 | 8 | 9 | 10 | 11 | 12 | 13 | 14 | 15 | 16 | 17 | 18 | 19 | 20 | 21 | 22 | 23 | 24 | 25 | 26 | 27 | 28 | 29 | 30 | 31 | ³32 |
|  |  | **Zone diameter (mm)** | | | | | | | | | | | | | | | | | | | | | | | | | | |
| **Supplementary Figure 2: Scattergram of MICs versus zone diameters for tylosin.** Isolates in bold were confirmed by PCR to have the resistance determinant *ermB* and those that are underlined to have the resistance determinant *msrC.* | | | | | | | | | | | | | | | | | | | | | | | | | | | | |

**References**

Naser, S.M., Fabiano, L., Thompson, F. L., Hoste, B., Gevers, D., Dawyndt, P., Vancanneyt, M., and Swings, J. (2005a). Application of multilocus sequence analysis (MLSA) for rapid identification of *Enterococcus* species based on *rpoA* and *pheS* genes. *Microbiol.*151, 2141-2150.

Naser, S., Thompson, F.L., Hoste, B., Gevers, D., Vandemeulebroecke, K., Cleenwerck, I., Thompson, C.C., Vancanneyt, M., and Swings, J. (2005b). Phylogeny and identification of Enterococci by *atpA* gene sequence analaysis. *J. Clin. Microbiol.*43, 2224-2230.

Tsai, J.C., Hsueh, P.R., Lin, H.M., Chang, H.J., Ho, S.W., Teng, L.J. (2005). Identification of clinically relevant *Enterococcus* species by direct sequencing of *groES* and spacer region. J. Clin. Microbiol. 43, 235-241.

Zaheer, R., Cook, S. R., Klima, C. L., Stanford, K., Alexander, T., Topp, E., Read, R. R., and McAllister, T. A. (2013). Effect of subtherapeutic vs. therapeutic administration of macrolides on antimicrobial resistance in Mannheimia haemolytica and enterococci isolated from beef cattle. *Front. Microbiol.* 4, 133-133.
